# Coupling a Federal Minimum Wage Hike with Public Investments to Make Work Pay and Reduce Poverty

**DOI:** 10.7758/rsf.2018.4.3.02

**Published:** 2018-02

**Authors:** Jennifer Romich, Heather D. Hill

**Affiliations:** School of Social Work at the University of Washington.; Daniel J. Evans School of Public Policy and Governance at the University of Washington.

**Keywords:** minimum wage, income support, poverty

## Abstract

For more than a century, advocates have promoted minimum wage laws to protect workers and their families from poverty. Opponents counter that the policy has, at best, small poverty-reducing effects. We summarize the evidence and describe three factors that might dampen the policy’s effects on poverty: imperfect targeting, heterogeneous labor market effects, and interactions with income support programs. To boost the poverty-reducing effects of the minimum wage, we propose increasing the federal minimum wage to $12 per hour and temporarily expanding an existing employer tax credit. This is a cost-saving proposal because it relies on regulation and creates no new administrative functions. We recommend using those savings to “make work pay” and improve upward mobility for low-income workers through lower marginal tax rates.

The first decade and a half of the twenty-first century have seen considerable changes in state and local minimum wages alongside calls for parallel changes at the federal level. Well over half the population now lives in areas subject to wage floors above the federal minimum of $7.25 per hour (author calculations). Because almost 60 percent of poor households headed by adults age eighteen to sixty-four include at least one employed person, higher wage mandates are designed to lower poverty and close the poverty gap among the working poor (U.S. Census Bureau 2015a). Indeed, minimum wage advocates argue for higher wages in part based on anti-poverty effects. Despite the seeming promise of anti-poverty effects, scholarly evidence suggests a modest effect of minimum wage increases on poverty rates. In this article, we set out to understand how the goals of the minimum wage—to reduce poverty and make work pay—might be better realized.

We begin with a brief history of the minimum wage in the United States, review what is known about its impact on poverty, and describe several explanations for relatively modest estimates of the effects of minimum wage laws on poverty. As others have noted, the minimum wage is imperfectly targeted to benefit poor or near-poor workers, which may dampen the effects of the policy on poverty. In addition, evidence suggests some offsetting effects of the minimum wage on earnings and employment (see, for example, Neumark and Wascher 2007; Belman and Wolfson 2014). We introduce a third explanation for muted effects on poverty: Interactions between work earnings and a set of income support programs—including the Supplemental Nutrition Assistance Program (SNAP), the Earned Income Tax Credit (EITC), and housing and childcare subsidies—for low-income workers.

To boost the poverty-reducing effects of the minimum wage, we propose an increase of the federal minimum wage to $12 per hour combined with a temporary expansion of an existing employer tax credit program, the Work Opportunity Tax Credit (WOTC), designed to reduce disemployment effects of the minimum wage. This combination is intended to lift workers and their dependents out of poverty. We also suggest that the cost savings of this proposal be invested in reducing marginal tax rates for low-income workers to promote work and upward mobility.

## THE EVOLUTION OF U.S. MINIMUM WAGE LAWS

Modern American minimum wage laws have their roots in the Fair Labor Standards Act of 1938 (FLSA). Following the Great Depression, the FLSA was a key part of President Roosevelt’s agenda. The secretary of labor, Frances Perkins, carefully crafted the law to withstand Supreme Court scrutiny, which had previously blocked state and federal wage regulation measures (Grossman 1978). Passed by a reluctant Congress on the basis of strong public support, the FLSA established minimum wages ($0.25 per hour at passage, rising to $0.40 over the seven years after passage), capped work hours (forty-four hours per week decreasing to forty hours over three years), and abolished most child labor.

Source documents suggest that the intent of the FLSA was to better the well-being of workers, making it an important complement to the social insurance programs created a few years earlier by the Social Security Act (Armstrong 1932). In light of concerns over “wage slavery” and “sweatshops,” on the eve of signing the FLSA, Roosevelt remarked, “Except perhaps for the Social Security Act, [the FLSA] is the most far-reaching, the most far-sighted program for the benefit of workers ever adopted” (as quoted in Grossman 1978, 22). The preamble to the FLSA established its purpose as addressing “labor conditions detrimental to the maintenance of the minimum standard of living necessary for health, efficiency, and general well-being of workers” (U.S. Department of Labor 2011).

Racialized policies of the time tempered this liberal promise, however. As with other Progressive Era policy advances, exclusions in the FLSA reinforced native-born white economic interests at the expense of other groups (Fox 2012; Katz 1986). Like the Social Security Act, the original FLSA excluded agricultural and domestic work, sectors dominated by African American workers, particularly in the South (Davies and Derthick 1997; Palmer 1995). In establishing a higher minimum wage, the FLSA protected the wages of U.S.-born white men who would otherwise face wage-lowering competition from immigrants and other races (Leonard 2005). These exclusionary policies persisted a half century until substantially remedied during the Civil Rights era by modifications of the FLSA and court decisions.

The basic structure of the federal minimum wage established by the FLSA continued throughout the twentieth century with periodic increases in the wage and gradually more workers gaining coverage. At the time of its passage, the FLSA covered roughly one-fifth of the U.S. workforce; major industries, including railroads and most retailers, were exempt from some or all requirements (Grossman 1978). Amendments in the 1960s and 1970s extended coverage to major retailers, domestic workers, and many farm and service-sector employees (U.S. Department of Labor 2009b). More recent estimates indicate that more than 80 percent of workers are covered (Bureau of Labor Statistics 2010; U.S. Department of Labor 2009a). The federal minimum was also increased over a dozen times over the twentieth and early twenty-first centuries, to a nominal level of $7.25 by 2010. The real value of the minimum wage has declined since 1968, when increases in the minimum wage stopped keeping pace with inflation.

The FLSA sets a floor for covered workers’ wages but does not preempt higher state or local wage requirements. From the FLSA’s earliest days, a handful of states had minimum wage laws that set a wage rate higher than the FLSA stipulated, and the number has grown over time. In 1980, for instance, the federal minimum wage was $3.10 and three states had rates between $3.20 and $3.50. By 2010, the federal rate was $7.25 and thirteen states had rates ranging from $7.40 to $8.55. Until recently, the difference between the federal and state rates was relatively modest: in 2010, higher state rates were an average of 8.4 percent higher than $7.25 (U.S. Department of Labor 2014).

Beginning in the 2012, a groundswell of state and local policy action on minimum wages created two innovations. First, states increased their wage rates to much higher levels, in terms of absolute value and relative to the federal wage rate, than ever before. By 2016, thirty states and District of Columbia mandated super-federal minimum wages, ranging from $7.50 to $10.50, and averaging 21 percent above the federal minimum of $7.25 (U.S. Department of Labor 2014). Second, for the first time, cities and counties have created local minimum wages above their state minimum wages. Since 2012, at least forty-six localities have passed minimum wage laws calling for wage standards as high as $15 per hour. Overall, using population estimates from the U.S. Census Bureau (2015a), we estimate that 61 percent of the U.S. population now lives in a state, county, or city subject to a higher-than-federal minimum wage, and more increases seem likely over the next few years.

The scope and extent of increases to minimum wage laws at various levels suggest that this is an important area of policy change affecting the working poor. Local and state legislation, and the movement behind them, are now ahead of and putting pressure on national policymakers to increase the federal minimum wage. In part because of a vocal movement among fast food workers, $15 per hour has become a rallying cry and a policy goal. Just as the early advocates for the FLSA highlighted its potential to improve the economic circumstances of workers, the current advocates focus on the potential of minimum wages to reduce poverty and inequality.^1^

## EVIDENCE ON MINIMUM WAGE EFFECTS ON POVERTY

Existing research on the poverty-reducing effects of the minimum wage is mixed, but generally concludes that minimum wage increases are associated with neutral or modest negative (lowering) effects on poverty rates. Arindrajit Dube’s summary of twelve studies on this question concludes that, on average, a 10 percent increase in the minimum wage leads to a 1.5 percent reduction in the poverty rate (2017). This elasticity is about the same anti-poverty effect of the federal disability insurance program and slightly higher than that of unemployment insurance or the EITC (Ben-Shalom, Moffitt, and Scholz 2012). Critics call Dube’s conclusion optimistic, raising concerns that methodological choices in the underlying studies and that the studies reviewed focus on the experiences of subgroups most likely to be affected bias the conclusion (Sabia 2014). In addition, some evidence suggests that short-term reductions in poverty fade away or reverse in the long term (Neumark and Wascher 2002). Importantly, the evidence relies on relatively modest state variation prior to 2010; the more substantial state and local increases of late should soon yield new findings.

Choices about measuring income and the poverty line may also cloud findings across studies. Of the studies that Dube reviewed, as well as his own analysis (2017), eight use the official poverty measure (OPM) or something akin to it (Addison and Blackburn 1999; Burkhauser and Sabia 2007; Morgan and Kickham 2001; Neumark, Schweitzer, and Wascher 2005; Sabia 2008; Sabia and Burkhauser 2010; Stevans and Sessions 2001). Two additional studies do not include taxes or transfer income (Card and Krueger 1994; Neumark and Wascher 2011), and another two use one or the other, but not both (Gundersen and Ziliak 2004; Sabia and Nielsen 2015). Only one, Robert DeFina’s cross-state analysis of minimum wage rates and child poverty, includes cash transfers as well as both net taxes and in-kind transfers (2008).

The editors of this double issue outline the limits of the OPM in an earlier article (Berger, Cancian, and Magnuson 2018). These limitations, namely the exclusion of SNAP and EITC, mean that the OPM fails to capture some anti-poverty effects associated with wage increases. Although differences in samples and subgroups preclude drawing any easy conclusions about the relationship between measurement and findings about poverty, that research has estimated the impacts on poverty largely without considering major anti-poverty income support programs is striking. In effect, researchers and advocates may be setting the bar for the minimum wage unreasonably high: to reduce official poverty rates, the policy would have to increase wages enough that *earnings alone* bring families above the poverty line.^2^

Beyond measurement issues, we believe three interrelated factors, covered in turn in the following section, explain the modest poverty-reducing effects of the minimum wage: imperfect targeting, the heterogeneity of labor market outcomes, and interactions with income support programs.

## IMPERFECT TARGETING

Although minimum wages aim to reduce poverty, they do not target the poor or near-poor population as well as many means-tested transfer programs. Opponents of the minimum wage have long argued that minimum wage workers are disproportionately young and working part time. Proponents of the minimum wage counter that, relative to all workers, minimum wage workers are also disproportionately female, African American, and Hispanic, groups that have traditionally been disadvantaged in the labor force and who have higher rates of poverty. Both characterizations are factually correct: Just 3 percent of all employed persons are sixteen to nineteen years old, but 19 percent of those working at or below the minimum wage are that age. Similarly, 50 percent of minimum wage workers work part-time hours, compared to 15 percent of all those employed. Fifty-nine percent of workers at or below the federal minimum wage are female; 21 percent are Hispanic; and 13 percent are black, versus 47, 15, and 11 percent among adult workers, respectively (Belman, Wolfson, and Nawakitphaitoon 2015).

There is less evidence of the joint distribution of poverty and minimum wages. The two published analyses of the characteristics of minimum wage workers use the Current Population Survey (CPS) Outgoing Rotation Groups (ORGs), which do not include family income or poverty measures (Belman, Wolfson, and Nawakitphaitoon 2015; Bureau of Labor Statistics 2010). To describe the prevalence of poverty and near poverty among workers by wage, and the wage distribution among working poor and near-poor adults, we linked four CPS ORGs from March to June 2010 to the CPS Annual Social and Economic Supplement, which includes family income and poverty measures.^3^ Table 1 presents the percentage poor, near poor, and neither for all adults, all working adults, all hourly workers, and then by hourly wage rate. The national poverty rate for all adults is 12.5 percent, but that includes many adults who are not working due to unemployment, disability, or retirement. Among working adults, the poverty rate is much lower, 6.23 percent. An additional 5.78 percent of all adults and 7.6 percent of all working adults are in near-poor families with incomes between 100 and 150 percent of the federal poverty line.

Not surprisingly, the poverty and near poverty rates are higher among hourly workers than among all working adults, 8.47 and 8.02 percent, respectively. As we look across the wage distribution, poverty rates are highest among those hourly workers with wages between $7.26 and $10.15: 16.89 percent are poor and another 13.6 percent are near poor. Importantly, this group has higher poverty rates than the workers earning at or below the federal minimum wage (14.3 percent) and higher rates than those for all adults (12.5 percent; a group that includes unemployed, disabled, and retired individuals). For workers with wages between $10.16 and $12 per hour, the poverty rates are lower than the rates for workers at the current federal minimum wage rate, but the near poverty rates are higher (10.9 percent versus 7.45 percent). At wages above $12 per hour, poverty and near poverty rates are all comparable to the rates of all employed adults.

Table 1 also shows the wage distribution among adults who are poor, near poor, and neither (column percentages as indicated). Among poor adults, about one-third were working in the week before the survey. Of those, the vast majority (80 percent) are paid hourly. Among those paid hourly, 80 percent earn a wage below $12 per hour. Near-poor adults are more likely to be working (50 percent) than poor adults are, but equally likely to be paid by the hour, conditional on working. Most (73 percent) near-poor workers paid hourly are making less than $12 per hour.

In sum, although the minimum wage imperfectly targets poor and near-poor families, it does disproportionately benefit disadvantaged workers, including women and persons of color. In addition, increasing the minimum wage as high as $12 per hour would improve the targeting of this policy toward reducing poverty.

## HETEROGENEOUS LABOR MARKET OUTCOMES

Pretend for a moment that earnings are the only source of income for workers. For minimum wage workers who maintain employment, higher wage mandates result in mechanical increases in hourly cash pay. However, the overall effects of minimum wage increases on earnings (wage times hours worked) are more complex. Reductions in hours may offset, partially or fully, increased wages. Workers who lose their jobs (or are unable to find jobs) may experience flat or even lower earnings.

As summarized in table 2, relative to a counterfactual of a lower minimum wage, we can imagine then a higher minimum wage causing four possible employment and earnings outcomes: first, increased earnings for employed workers when the increased wage rate times hours worked is greater than any loss in hours worked; second, flat earnings when decreases in hours offset increased hourly pay; third, reduced earnings in the event that reductions in hours exceed the value of a new higher hourly pay; or, fourth, unemployment. These four possible outcomes are mutually exclusive for a given jobholder at one point in time, but almost certainly co-occur across a population, within two-worker families, and within workers over time.

The extant literature has paid most attention to the average population-level disemployment effects of state difference in minimum wage or in federal minimum wage increases. These studies primarily focus on teenagers and young adults, and on relatively small differences in minimum wage. One comprehensive review concludes that the majority of studies finds small but non-negligible disemployment effects (Neumark and Wascher 2007). A more recent meta-analysis concludes that raising the minimum wage 10 percent would lead to negative impacts on employment and hours in the range of 0 (null) to 2.6 percent (Belman and Wolfson 2014), a range consistent with other studies not included in the meta-analysis (for example, Neumark, Salas, and Wascher 2014). We know far less about the effects of recent state and local minimum wages, which are often much larger increases than those studied previously. Consistent with prior literature, early results from the evaluation of Seattle’s minimum wage ordinance suggest wage increases, small reductions in jobs, and a null to small increase in earnings depending on the comparison group (Seattle Minimum Wage Study Team 2016b).

Any net effect of a higher minimum wage on poverty depends not only on the distribution of affected workers across these four categories in table 2, but also on the distribution of labor market effects for workers with family income near the poverty line. Poverty rates should decrease if group one (with increased earnings) is made up of workers at or below the poverty line, but that benefit could be reduced or completely offset by workers above the poverty line falling into groups two or three (with decreased earnings). David Neumark and William Wascher find that the poverty-reducing benefits were reduced over time in this exact fashion (2002).

## INTERACTIONS WITH INCOME SUPPORTS

**Figure 1.**
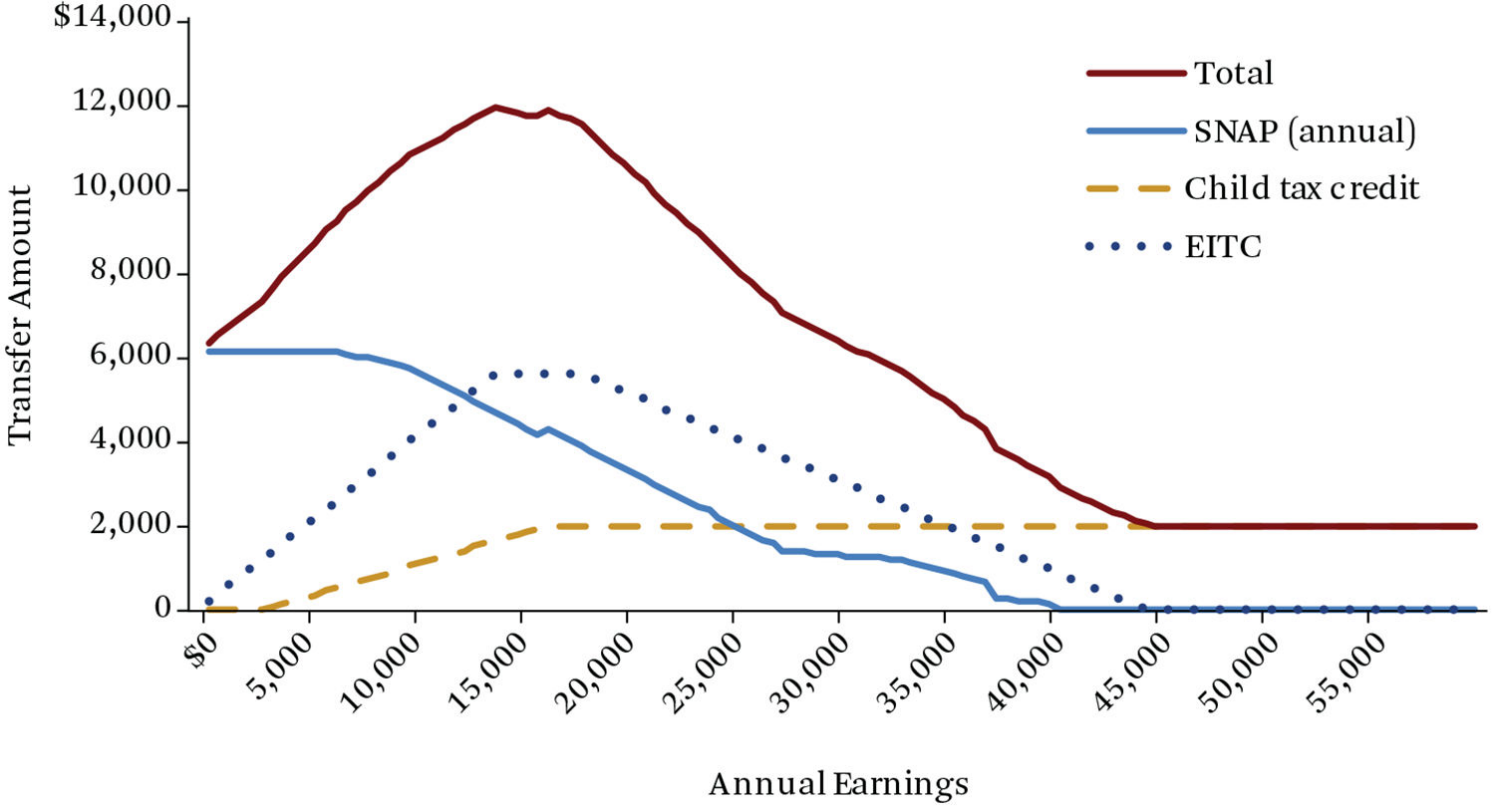
Annual Values of Income Support Transfers *Source:* Authors’ calculations (see online appendix for details).

**Figure A1.**
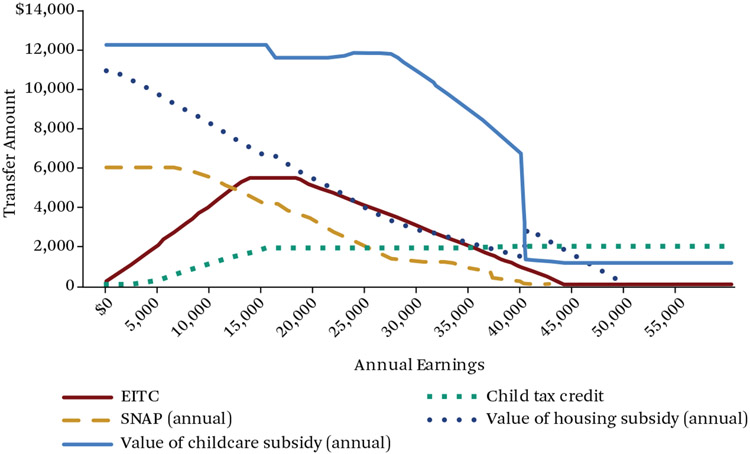
Five Common Income Support Transfers, One-Adult, Two-Child Household *Source:* Authors’ calculations. *Note:* Net federal taxes include Earned Income Tax Credit, Child Tax Credit, income tax liability and worker’s nominal portion of FICA payroll tax. Supplemental Nutrition Assistance Program (SNAP) benefits calculated based on average of calculators for three states. Housing subsidy calulated from U.S. Department of Housing and Urban Development rules for Section 8 program, assuming median U.S. rent of $920 per month. Childcare voucher calculated from Child Care Aware data on costs of childcare in the United States and Washington State child subsidy co-pays. Because part-time workers are unlikely to qualify for full-time care, assumed costs of childcare are 0.75 of full value. The Poverty Threshold (2015) is calculated by the Census and published each year.

In reality, earnings are not the only source of income. Low-income workers and their dependent children qualify for and receive public assistance in the form of cash transfers, commonly through the tax system; benefits with cash-like values, such as food assistance; and in-kind subsidies for goods and services such as housing and childcare. On average, the lowest income families in the United States receive 34 percent of overall income from government income support programs, versus 14 percent, on average, among all families (Congressional Budget Office 2016). Figure 1 shows the annual value of three common income supports—the EITC, the Child Tax Credit (CTC), and SNAP—for an exemplar family consisting of one adult and two dependent children at different earnings levels. We include these three federally funded programs because everyone who qualifies is entitled to benefits, they have high take-up rates, and they provide substantial benefits with little to no variation between different states. Figure A1 displays the same information but adds two common local- and state-administered programs, subsidized housing and childcare, highly valuable benefits that are subject to rationing.

SNAP provides the greatest benefits at low-income levels—more than $6,000 for a family of three in 2016—and phases out as earnings rise. The EITC phases in over low income levels, reaches a plateau maximum of $5,572 for a single earner with two children and $6,269 for joint filers with three children in 2016. Finally, the CTC, which adds to the value of the tax refund for low-income families and offsets taxes owed for most nonwealthy families, phases in as earnings rise to a maximum value of $1,000 per child. Together, these three transfers can total up well above $10,000 per year at earnings levels that correspond with part- or full-time work at the current federal minimum wage of $7.25 per hour. Publicly funded childcare and housing supplements—as shown in figure A1—can add an additional $15,000 or more of in-kind value, due largely to the high market cost of these goods. Unlike tax credits and SNAP, however, childcare and housing subsidy slots are limited. Approximately one in four eligible households receives federal housing subsidies; fewer than one in six potentially eligible children have care funded through the Child Care Development Block Grant; and presumably much lower fractions of eligible families receive both benefits (Chien 2015; Congressional Budget Office 2015).

The phase-out of the combined benefits, beginning around $15,000 of annual earnings, shows that households will experience partially offsetting declines in transfer payments or in-kind assistance as incomes rise. These interactions between earnings and means-tested income supports—known as implicit marginal tax rates (MTRs)—are the result of the eligibility and benefit rules necessary to target assistance to economically disadvantaged families (Romich 2006). Moderate to high MTRs are likely to lessen or even thwart the well-being enhancing effects of the minimum wage. Of course, replacing transfer income with earned income likely appeals to both policymakers—who generally want to restrict public expenditures—and workers. For workers, cash earnings are more flexible than SNAP benefits because they can be spent on anything, and earnings are more immediate and intuitive than tax credits, which arrive in a lump sum only once per year. Finally, earned income rather than transfers may enhance dignity and reduce stigma, although recipients report little stigma from the substantial EITC and CTC payments that come in the form of tax refunds (Levin et al. 2015).

The limited literature on the impacts of higher minimum wages on means-tested benefit use largely parallels the poverty findings of modest to no effects and is consistent with the phase-out pattern noted here. Michael Reich and Rachel West find that higher minimum wages reduced SNAP enrollment (2015). They estimate an increase in the federal minimum wage from the current $7.25 to $10.10 would decrease total SNAP enrollment by about 8 percent. Preliminary work from Joseph Sabia and Thanh Nguyen examines SNAP, energy assistance and Medicaid, and finds null to modestly negative impacts of higher minimum wages on program receipt (2015). Importantly, these studies are not examining whether the overall economic well-being of the family has improved, declined, or stayed the same relative to the counterfactual.^4^

In service of a more robust understanding of interactions between minimum wage levels and means-tested benefits, we calculated family disposable income relative to the official poverty threshold for eight family types at four different minimum wage levels (table 3). The family types are one parent and zero to three children, and two parents and zero to three children. The four wages are the current federal rate of $7.25; the current federal rate for federal contractors of $10.15; and $12 and $15, which have been proposed or established in some states and cities. Full- and part-time work consist of two thousand and one thousand hours per year, respectively.

While we are using the OPM threshold, we combine income sources to get closer to the Supplemental Poverty Measure’s definition of income.^5^ We start with earnings only, add taxes and SNAP, and finally add childcare and housing subsidies. We consider the “earnings plus taxes and SNAP” income to be the best measure of income for workers earning low wages because of the high take-up rates of both the EITC and SNAP. Although fewer workers receive childcare and housing subsidies, they are substantial income supports for recipients. Importantly, these are static models, in which we assume no disemployment effects. Tables A1 and A2 display the income values (instead of income relative to poverty) and the online appendix details our assumptions for these calculations.

**Figure 2.**
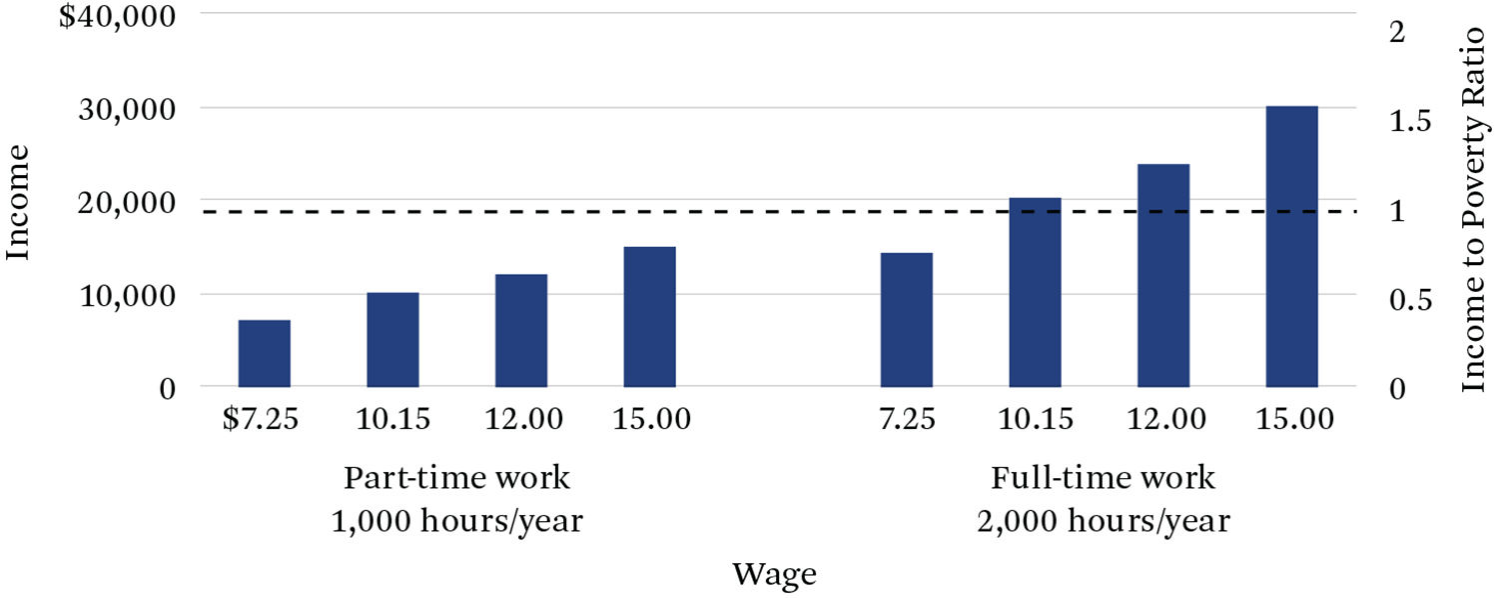
Earnings *Source:* Authors’ calculations (see online appendix for details).

**Figure 3.**
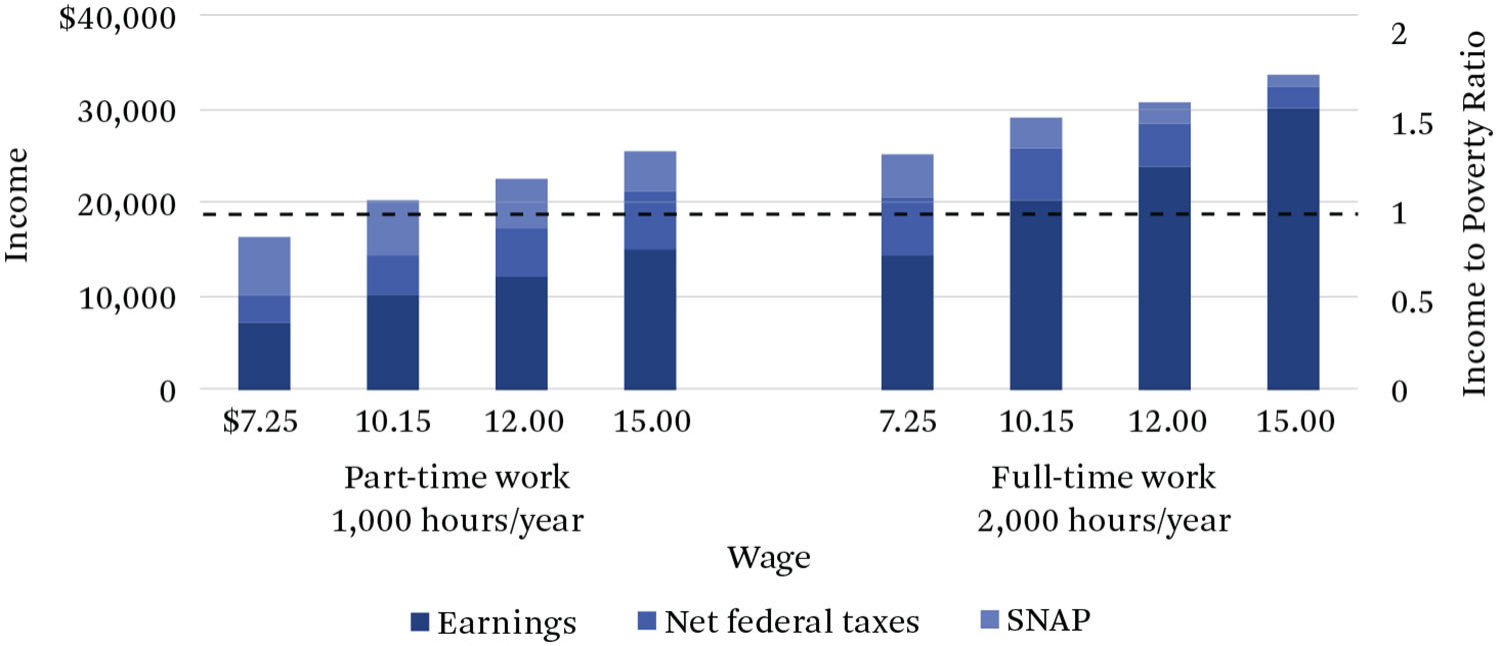
Earnings, Net Federal Taxes, SNAP *Source:* Authors’ calculations (see online appendix for details). *Note:* Figures are earnings plus taxes and income support transfers at different minimum wage levels; one adult, two-child household, 2016 tax and benefit amounts. Net federal taxes include Earned Income Tax Credit, Child Tax Credit, income tax liability and worker’s nominal portion of FICA payroll tax. Supplemental Nutrition Assistance Program (SNAP) benefits calculated based on average of calculators for three states. The Poverty Threshold (2015) is calculated by the census and published each year.

**Figure 4.**
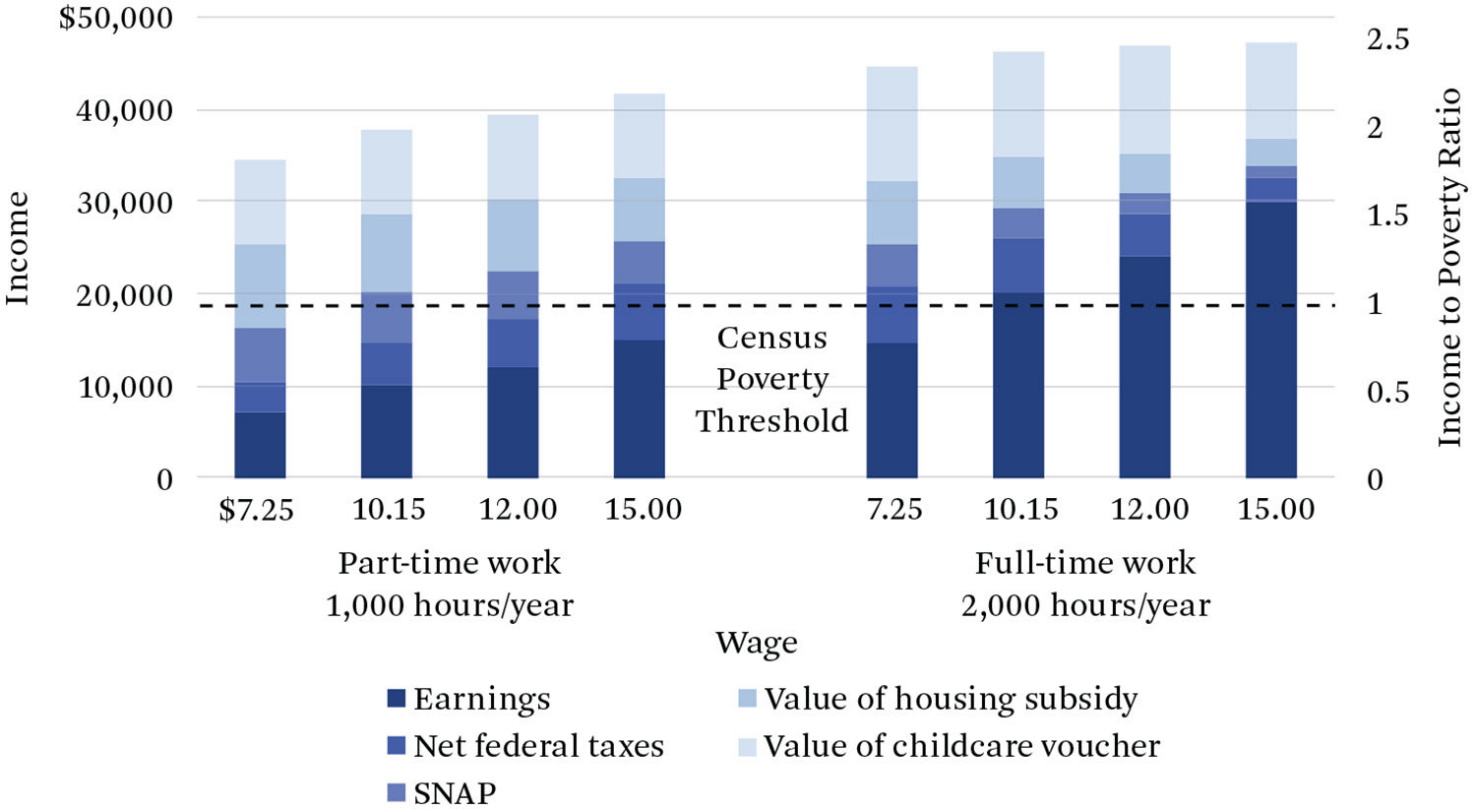
Earnings, Net Federal Taxes, SNAP, Housing, and Childcare Assistance *Source:* Authors’ calculations (see online appendix for details). *Note:* Figures are earnings plus taxes and income support transfers at different minimum wage levels; one adult, two-child household, 2016 tax and benefit amounts. Net federal taxes include Earned Income Tax Credit, Child Tax Credit, income tax liability and worker’s nominal portion of FICA payroll tax. Supplemental Nutrition Assistance Program (SNAP) benefits calculated based on average of calculators for three states. Housing subsidy calculated from U.S. Department of Housing and Urban Development rules for Section 8 program, assuming median U.S. rent of $920 per month. Childcare voucher calculated from Child Care Aware data on costs of childcare in the United States and Washington State child subsidy co-pays. Because part-time workers are unlikely to qualify for full-time care, assumed costs of childcare are 0.75 of full value. The Poverty Threshold (2015) is calculated by the census and published each year.

Figures 2, 3, and 4 display the results graphically for the exemplar household of a single adult with two children under age seventeen. Two important aspects of this discussion are displayed in figures 2 through 4: the extent to which minimum wage earnings—alone or in conjunction with means-tested benefits—raise families above the official poverty line; and the extent to which part-time versus full-time work effort and minimum wage increases pay off, that is, does income increase proportionally to earnings? The key findings from this figure are generally true for all the family types shown in tables 3 and 4.

In figure 2, earnings alone raise the family above the poverty line only when full-time work is paired with a wage around $12 per hour. Even at $15 per hour (the highest minimum wage currently under widespread consideration), half-time work does not raise the family above poverty. When we consider earnings alone, full-time work pays more than half-time work, and earnings clearly rise with increases in the minimum wage. These are mechanical effects, as the top panel does not include any explicit or implicit taxes.

The combination of higher minimum wages with the two major income support programs (EITC and SNAP) is what successfully raises family resources above the poverty line. Figure 3 shows earnings plus SNAP benefits and net taxes, which includes the EITC and CTC minus the employee portion of payroll taxes and any federal income tax liability. When the value of SNAP and tax credits are figured in, full-time minimum wage work at the current federal rate of $7.25 raises one or two adult families with up to three children above the poverty line, as does half-time work at the $10.15 rate or above. Figure 3 displays the addition of publicly funded childcare and housing assistance, two locally administered, federally funded programs subject to rationing. For families who qualify for and receive these two benefits, full- or part-time work at any level would raise them above the poverty line.

In addition, the middle and bottom panels show the flattening of income (post-tax and transfer income) with each wage step because of increasing marginal tax rates. MTRs are the combination of payroll and income tax rates, as well as the implicit tax rates associated with means-tested benefit reductions. Tables A3 and A4 in the online appendix summarize the MTRs for each family type, for part- and full-time workers, and for two combinations of income supports when moving from one wage level to the next. Depending on the family size, wage rate, and income supports received, marginal tax rates can range from −41 percent to nearly 100 percent.

Two primary factors are associated with marginal tax rates over 50 percent: full-time hours and receiving the combination of EITC, SNAP, housing subsidies, and childcare subsidies. Overall, part-time work hours produce low or even negative marginal tax rates, particularly for families with children. This is the result of these families still being on the phase-in portion of the EITC schedule, which means that each additional $1 earned results in $1.30 to $1.45 of income. The MTRs are not as low for part-time workers *without children* because the phase-in for the childless EITC is only a 7 percent wage subsidy. In contrast, full-time hours place most families in the phase-out portion of the EITC schedule, meaning that the benefit falls with every additional dollar earned. SNAP benefits phase out as well, leading to MTRs of 22 to 41 percent for full-time workers moving from $7.25 to $10.15, depending on the number of children. The MTRs for full-time workers increase to as much as 60 percent for those moving to the $12 and $15 level. Each additional dollar earned with minimum wage increase would still raise disposable income, but by far less than earnings increase.

The combined phase-out of EITC, SNAP, housing, and childcare yields especially high MTRs. For full-time workers, moving from $7.25 per hour to $10.15 per hour would result in an effective MTR of 69.3 percent, and the earnings increase from $12 to $15 per hour would yield almost no effective increase in disposable income, with an MTR of 94.9 percent.^6^ Such low yield from an additional dollar in earnings stands in opposition to the “work pays” social contract of the late twentieth and early twenty-first century. This can be seen most clearly in figure 4, where working full-time hours at $15 per hour produces roughly the same annual income as working part-time hours at that same wage rate. High MTRs can create a disincentive to work more hours or at better wages, which could stifle upward mobility. Moreover, qualitative studies find that families subject to high MTRs find them demoralizing and destabilizing (Romich 2006).

## THE PROPOSAL: A $12 FEDERAL MINIMUM WAGE PLUS TARGETED PUBLIC INVESTMENTS

It is our belief that full-time work hours should raise individuals and families above poverty, and that part-time work hours combined with SNAP and EITC should do the same. In addition, low-income families should not have wage increases offset entirely by explicit or implicit taxes. In other words, work should pay. To achieve these goals, we propose to raise the federal minimum wage to $12 and index it to inflation, and to expand temporarily the Work Opportunity Tax Credit to mitigate disemployment effects during the transition to the higher wage. This is a cost-saving proposal because it relies largely on regulation and does not create new programs or administrative structures. We recommend using the savings to adjust payroll tax rates and the phase-outs of income supports to keep marginal tax rates at 50 percent or lower up to 200 percent of the poverty line. In the following sections, we describe and support each part of the proposal.

### Minimum Wage Increase

We propose raising the federal minimum wage to $12 per hour, with a two-year phase-in, and to index it to inflation moving forward. As our simulations show, at this wage level, a full-time worker with a spouse and two children will earn enough to be above the poverty line (the same is true if both spouses work part-time hours that sum to full-time work). When combined with SNAP and federal tax credits net of payroll taxes, a half-time single worker with three or fewer children will be nonpoor as well. We believe that half-time work is an appropriate expectation for low-wage workers whose jobs disproportionately feature irregular and unpredictable hours (Tilly 1991; Lambert, Fugiel, and Henly 2014), particularly parents who must balance market work and family caregiving. Indeed, Luke Shaefer estimates that 36 percent of all part-time workers are primary earners for their families (2009).

Indexing the federal minimum wage to the cost of living is critical to its success. In fact, the decline in the real value of the minimum wage over time has greatly exacerbated concerns about disemployment. With a minimum wage designed to increase proportionally with changing costs or wages in this country, we would not face the need to increase the wage by such a large amount in future years. Several states have recently passed laws or voter initiatives that index the state minimum wage to inflation.

We recommend that the increase from $7.25 to $12 take effect over two years without mandated steps. This time and flexibility will maximize employers’ ability to make decisions about when and how much to increase wages as to best absorb the increased personnel costs. Some recent local and state laws mandate structured steps across many years, but we worry that this approach is overly complicated and might make enforcement more difficult.^7^

The size of this increase relative to current minimum wages would vary considerably by state and even city. By our calculation, the increase would be below some city-level wage mandates but up to 60 percent above the current laws across the country. The larger increases in places that adhere to the federal minimum wage are beyond any recent experience or evidence. As we describe in the following section, some of the public investments that accompany the minimum wage increase could be targeted directly to states and localities facing particularly large increases.

### Temporary Expansion of the Work Opportunity Tax Credit

From our perspective, there is enough evidence on modest state and federal minimum wage increases to suggest that higher state minimum wages can lead to small reductions in employment. Targeted public investments could shore up employment in the private, nonprofit, and public sectors. Although the debate about the minimum wage often focuses entirely on the reaction of employers in the private sector, public and nonprofit organizations may be most vulnerable (Allard 2016). Both nonprofits and public organizations do not have profit margins to dip into to shift wages up, they are often underfunded as it is, and many serve exactly the population that the minimum wage is designed to benefit.

We propose offering temporary subsidies to employers of low-wage workers to support their absorption of higher personnel costs in the transition period. This could be done at reasonably low cost through the existing Work Opportunity Tax Credit, a $1 billion program, offering tax credits between $1,200 and $9,600 per year for each worker hired from a target group, including veterans, SNAP recipients, and ex-felons. The program is also available to nonprofits through offsets to the employer portion of payroll taxes. We recommend a two-year expansion of the WOTC program with these four parameters:

Double the overall size of the budget available to $2 billion for each of the two years of the transition period to the higher minimum wage.

Allow the credit to be claimed for not only hiring, but also retaining employees at a higher wage.

Maintain the current target groups, but add an additional group (temporarily) of workers making at or less than $15 per hour.

Increase the maximum credit per employee for employers from *maximally affected* industries or geographic locations. Maximally affected would be defined as nonprofit, small businesses, and any business in a location experiencing a minimum wage increase of 25 percent or more.

The limited research evidence on WOTC suggests that it boosts worker earnings but reduces job tenure in the short term (Hamersma and Heinrich 2007). Some researchers have argued that it is a windfall for employers who would have hired the targeted employees without the subsidy (Lower-Basch 2011). Acknowledging these limitations, we still contend that WOTC is an effective way of supporting employers during the transition to a higher minimum wage. The temporary support would allow employers to take a longer, strategic view of how to shift their wage distribution without cutting jobs or hours. Subsidized employment (Dutta-Gupta et al. 2018) or a federal job guarantee (Paul et al. 2018) could achieve similar results while also developing human capital for those who might struggle in the private labor market, but both approaches are costlier than employer tax credits.

## COST AND EFFECT ESTIMATES OF THE PROPOSAL

This proposal for a $12.00 minimum wage combined with a temporary expansion of the WOTC is included in the common cross-chapter simulations described elsewhere in this double issue (Wimer, Collyer, and Kimberlin 2018). For the purposes of the cross-chapter simulation exercise, we stop short of a full dynamic model and examine only two outcomes: earnings and disemployment. For simulation purposes, we posit that 6.5 percent of workers making below the new minimum will become unemployed and that the remainder will receive raises. This amounts to a labor demand elasticity of about −0.1 relative to the 66 percent increase that $12 represents to the current federal minimum wage (although the actual increase would be lower in the many states that already have higher rates). This is within the range of disemployment effects predicted in the literature and a larger disemployment effect than assumed in prior simulations (Sawhill and Karpilow 2014; Congressional Budget Office 2014).^8^ Because we also model a substantial increase in the WOTC designed to support labor demand, we think this is a reasonable and conservative assumption. The simulation also accounts for changes in SNAP eligibility and federal tax liability and credits (as outlined in Wimer, Collyer, and Kimberlin 2018).

Simulation results suggest that a federal $12 minimum wage will lead to a 16.1 percent drop in poverty when measured by the SPM, from 14.3 percent to 12.0 percent. In elasticity terms, this means a 10 percent increase in the minimum wage leads to a 2.5 percent decrease in poverty, a figure that is 1 percentage point higher than found in Dube’s cross-study analysis based on prior, smaller increases (2017). Consistent with our observations on measurement, results using the OPM are slightly more modest. Increasing the minimum wage will also lead to net savings of $19.3 billion for the federal government, largely driven by increases in FICA revenue and net federal taxes, including a $4.4 billion decrease in EITC outlays.

## REDUCING MARGINAL TAX RATES FOR LOW-INCOME WORKERS

Although the changes modeled for the simulation exercise focus mainly on poverty reduction, additional investments could help stabilize low-income working families and make work pay as earnings increase. The net cost savings of the minimum wage increase and WOTC expansion means these investments can be made and still achieve a revenue-neutral intervention. These investments would be targeted at families with one or more workers and family income in the lower- and middle-income quintiles. They are designed to provide paths out of poverty and in to the middle class and to remove barriers to upward mobility.

As noted, relying on means-tested benefits and wages at or around the $12 per hour level creates high effective marginal tax rates for workers with dependent children. Reducing MTRs requires a tailored approach for each means-tested income support program by family size and other factors. Space limitations prohibit a fully specified proposal here, but we offer a few possible directions. One option to address the MTRs caused by the EITC phase-out would be to reduce the EITC phase-out rate below the current 40 percent, or to alter some other parameter of the tax system to achieve a similar result. Recent proposals to expand the EITC or CTC for certain groups range in cost from $10 to $25 billion per year. Changes to the EITC could increase its anti-poverty effect (Sawhill and Karpilow 2014). Increasing the CTC would target benefits to families in the lower- and middle-income quintiles, offsetting high MTRs (Maag 2016). Changing the withholding system or creating a periodic payment structure to deliver a portion of the credit to families throughout the year rather than just at tax time could further enhance well-being.

Alternatively, these funds could be used to expand key in-kind supports. Housing and childcare programs provide critical supports to the families that receive them, but they are both heavily rationed and have some of the steepest phase-out rates of any income support programs. Although both housing and childcare fill crucial needs, concerns about total cost and dependency are less with childcare, which serves a dual purpose of transfer and human development and has a natural sunset point when children age out of the need for paid care. In recent years, the federal government and states combined have spent $8.5 billion to serve 1.41 million children eligible for childcare subsidies via the Childcare and Development Block Grant (CCDBG) program (Administration for Children and Families 2017) but this is a fraction of eligible potential recipients. Recent estimates suggest that CCDBG funds reach less than 15 percent of eligible children (Chien 2015). Offsetting federal savings from a higher minimum wage by tripling the size of CCDBG with federal funds could serve many more families. With less rationing, states could direct more funds to families above the poverty line but below the CCDBG cap of 85 percent of state median income, the families who would face the highest combined effective marginal tax rates due to the phase-out of the EITC and SNAP.

## CONCLUSION

Minimum wage policies and income support programs emerged during the New Deal from the same impetus: protecting workers from economic deprivation. A wave of recent and proposed state and local minimum wage increases more explicitly aim to reduce poverty and inequality. We believe that a higher minimum wage can stand alongside other anti-poverty measures and have proposed a $12 federal minimum wage augmented by temporary public investments to reduce disemployment and marginal tax rates. Increasing the wage to $12 ensures that earnings alone lift a full-time worker and their family above poverty, and that earnings combined with the EITC and SNAP will do the same for part-time workers. In addition, a minimum wage of $12 better targets poor workers than our current minimum wage. We propose that the modest disemployment effects associated with minimum wage increases can be reduced through an employer tax credit for hiring and retaining workers at or near the minimum wage. The existing WOTC program is ideally suited to this purpose and could be further modified to direct these resources to employers who are particularly exposed to increased costs.

Symbolically, a higher minimum wage aligns strongly with a “work pays” ideal, encouraging employment as the primary means of avoiding poverty. Our calculations, however, point to the fact that just above the poverty line workers receiving higher wages and major income support, such as EITC and SNAP, experience high marginal tax rates. Workers that also receive childcare and housing subsidies can end up with marginal tax rates close to 90 percent, such that they keep only $0.10 of each additional $1.00 increase in wage. These high marginal tax rates do not affect poverty per se, but they reduce the return to working, cause frustration and demoralization, and create a potential barrier to upward mobility. We recommend using the cost savings from the minimum wage proposal to lower marginal rates below 50 percent for all low-income families through changes to federal taxes or means-tested income supports.

The racialized history of early minimum wage efforts make it important to explicitly consider the likely effects of this policy proposal on persons of color. Blacks and Hispanics are overrepresented among workers making the minimum wage or just above it (Belman, Wolfson, and Nawakitphaitoon 2015), suggesting that these workers will experience disproportionate benefit or harm from this policy. The Wimer and colleagues simulation results show that a $12 minimum wage will produce proportionally greater reductions in black and Hispanic poverty relative to white poverty (2018). Disemployment effects, however, may also hit these populations more acutely, particularly in light of documented racial discrimination in hiring (Pager, Western, and Bonikowski 2009).

The effects on families with children also merit particular attention. Raising the minimum wage can increase the proportion of poor and near-poor families’ incomes that is earnings rather than transfer income. Earnings may be more volatile than transfer income, which typically does not change other than during annual recertification periods, and income instability can be harmful for child development (Gennetian et al. 2015; Hardy 2014; Romich and Hill 2017). Alternatively, the high MTRs associated with benefit phase-out makes part-time work almost as lucrative as full-time work, possibly altering parents’ labor supply decisions. Part-time employment may help parents balance work and family responsibilities, and has been shown in national data to benefit children, relative to no work or full-time work, in the first year of life (Brooks-Gunn, Han, and Waldfogel 2010; Buehler and O’Brien 2011). Finally, because the childcare sector relies on low-wage workers, families who pay for childcare fully out-of-pocket may face increased costs.

This policy proposal entails some key trade-offs. Higher wages will do nothing to improve the lot of nonworkers, and even modest decreases in labor demand could increase the rate of economic disconnection, not being employed or on public assistance, although our simulation shows deep poverty lessening slightly. It is important that the increase we propose, and many of the local and state increases under way, are much larger percentage increases in the wage floor than any research has tested. We are in largely unknown territory as to the effects of 25 percent or larger wage increases on employment, earnings, income, and poverty. Politically, minimum wage increases garner strong opposition from organized business groups, particularly the restaurant industry and other sectors with large low-wage labor forces. However, they also have the political advantage of shoring up income without direct public expenditure.

The real value of the federal minimum wage has fallen too far below the cost of living, exacerbating poverty and inequality in the United States. Many states and cities have responded by passing minimum wages above the federal rate. These local laws will cover many workers, but they do not establish, symbolically or actually, a floor below which we, as a country, believe wages should not fall. The increase we propose can reset that federal floor and keep it from falling in the future. In addition, a higher federal minimum wage coupled with public investments will better target poverty than our current rate, and disproportionately benefit workers disadvantaged by race, gender, or geographic location. In sum, coupling a federal minimum wage increase with targeted public investments is an essential next step to reducing poverty and making work pay.

## Supplementary Material

Appendix

## Figures and Tables

**Table 1.** Income Relative to Poverty Among Adults

	Below 100	100 to 150	Above 150
**Adults**	12.5	7.6	79.9
Employed	6.23	5.78	87.99
Column percent	33.09	50.44	74.02
Paid hourly	8.47	8.02	83.51
Column percent	78.94	79.87	54.03
Wage Rate			
<=$7.25	14.3	7.45	78.25
Column percent	13.43	9.52	4.39
$7.26–$10.15	16.89	13.6	69.51
Column percent	55.62	47.05	23.93
$10.16–$12.00	7.01	10.9	82.09
Column percent	10.59	16.82	12.39
$12.01–$15.00	5.01	6.26	88.73
Column percent	10.14	14.4	18.28
$15.00+	2.36	2.56	95.08
Column percent	10.22	12.21	41

*Source:* Authors’ calculations based on the CPS (Flood et al. 2017).

*Note:* All estimates are weighted by either the ASEC weight (poverty rates) or the earner study weight (wages). Employment status, hourly pay, and wage rate were asked for the week prior to the survey. Poverty rate is based on the prior year’s annual income, family size, and the Federal Poverty Guidelines.

**Table 2.** Heterogeneous Effects of Increased Minimum Wage on Earnings

Possible Employment and Earnings Outcomes	Likely Change in Earnings-to-Poverty Ratio
Employed, earnings increase	Up
Employed, earnings flat	Unchanged
Employed, earnings down	Down
Unemployed	Down

*Source:* Authors’ calculations.

**Table 3.** Income to Poverty Ratios, One-Adult Households

	One Part-Time Worker				One Full-Time Worker			
Hourly Wage	7.25	10.15	12	15	7.25	10.15	12	15
**No children**								
Annual earnings	0.59	0.82	0.97	1.22	1.18	1.65	1.95	2.43
+ Taxes	0.58	0.79	0.9	1.09	1.05	1.44	1.67	2.05
+ SNAP	0.76	0.93	1.01	1.14	1.11	1.45	1.67	2.05
+ Housing and childcare subsidies	1.48	1.58	1.61	1.67	1.65	1.85	1.98	2.21
**One child**								
Annual earnings	0.44	0.62	0.73	0.92	0.89	1.24	1.47	1.84
+ Taxes	0.6	0.84	0.95	1.12	1.09	1.39	1.56	1.82
+ SNAP	0.86	1.08	1.15	1.29	1.27	1.48	1.62	1.84
+ Housing and childcare subsidies	1.75	1.92	1.96	2.01	2.11	2.23	2.24	2.19
**Two children**								
Annual earnings	0.38	0.53	0.63	0.79	0.76	1.06	1.26	1.57
+ Taxes	0.54	0.76	0.9	1.11	1.08	1.35	1.49	1.7
+ SNAP	0.85	1.06	1.17	1.34	1.32	1.53	1.62	1.77
+ Housing and childcare subsidies	1.81	1.97	2.06	2.18	2.33	2.42	2.45	2.47
**Three children**								
Annual earnings	0.3	0.42	0.5	0.62	0.6	0.84	1	1.24
+ Taxes	0.44	0.62	0.74	0.91	0.89	1.13	1.25	1.42
+ SNAP	0.76	0.93	1.03	1.16	1.15	1.33	1.42	1.52
+ Housing and childcare subsidies	1.66	1.79	1.86	1.96	2.13	2.22	2.26	2.29

*Source:* Authors’ calculations using 2015 Poverty Thresholds (U.S. Census Bureau 2017).

*Note:* Ratios are earnings plus taxes and income support transfers at different minimum wage levels. Thresholds vary by household size and number of children as follows: $12,331 (one adult), $16,337 (one adult, one child), $19,096 (one adult, two children), $24,120 (one adult, three children), $15,871 (two adults), $19,078 (two adults, one child), $24,036 (two adults, two children), $28,286 (two adults, three children).

**Table 4.** Income to Poverty Ratios, Two-Adult Households

	One Full-Time Worker				One Full-Time + One Part-Time Worker			
Hourly Wage	7.25	10.15	12	15	7.25	10.15	12	15
**No children**								
Annual earnings	0.91	1.28	1.51	1.89	1.37	1.92	2.27	2.84
+ Taxes	0.87	1.18	1.38	1.69	1.26	1.71	2	2.45
+ SNAP	1.04	1.25	1.4	1.69	1.31	1.71	2	2.45
+ Housing and childcare subsidies	1.46	1.56	1.64	1.82	1.59	1.83	2.01	2.45
**One child**								
Annual earnings	0.76	1.06	1.26	1.57	1.14	1.6	1.89	2.36
+ Taxes	0.93	1.21	1.39	1.6	1.28	1.62	1.81	2.12
+ SNAP	1.17	1.37	1.49	1.63	1.44	1.69	1.87	2.12
+ Housing and childcare subsidies	1.53	1.65	1.72	1.77	1.95	2.01	2.01	2.12
**Two children**								
Annual earnings	0.6	0.84	1	1.25	0.9	1.27	1.5	1.87
+ Taxes	0.86	1.09	1.23	1.41	1.15	1.42	1.56	1.79
+ SNAP	1.12	1.29	1.39	1.5	1.34	1.52	1.63	1.82
+ Housing and childcare subsidies	1.41	1.51	1.56	1.59	1.91	1.97	1.97	1.93
**Three children**								
Annual earnings	0.51	0.72	0.85	1.06	0.77	1.08	1.27	1.59
+ Taxes	0.76	0.98	1.11	1.26	1.03	1.27	1.4	1.6
+ SNAP	1.03	1.2	1.3	1.39	1.25	1.41	1.49	1.65
+ Housing and childcare subsidies	1.28	1.39	1.45	1.48	1.85	1.92	1.94	1.96

*Source:* Authors’ calculations using 2015 Poverty Thresholds (U.S. Census Bureau 2017).

*Note:* Ratios are earnings plus taxes and income support transfers at different minimum wage levels. Thresholds vary by household size and number of children as follows: $12,331 (one adult), $16,337 (one adult, one child), $19,096 (one adult, two children), $24,120 (one adult, three children), $15,871 (two adults), $19,078 (two adults, one child), $24,036 (two adults, two children), $28,286 (two adults, three children).

## Footnotes

1. Importantly, these are not the only arguments in favor of a minimum wage, but they are the arguments that motivate our consideration of the anti-poverty effects of the policy.

2. In addition, because of heterogeneous labor market effects and the phase-out issues noted later, failing to include the most common means-tested benefits would likely bias the estimates of the minimum wage’s impacts on poor- and near-poor families’ income. The bias could be upward, if marginal tax rates are very high and offset increased earnings, or if those who lose jobs or hours rely more on income support than they would have otherwise. The bias could also be downward, if the poverty-reducing effects of the minimum wage are entirely among those who combine higher earnings and income support (for example, families on the upward slope of the EITC schedule). To illustrate this point, consider the part-time and full-time worker scenarios depicted in panel B of figure 2. A part-time minimum wage worker moving from $7.25 to $12.00 per hour would still be poor under the OPM, but at the higher wage level, the household’s disposable income would be above the poverty line. In this case, analysis using the OPM would understate the anti-poverty effect. On the other hand, earnings plus SNAP and tax benefits raise a full-time worker above the poverty line at either $7.25 or $12.00 per hour, but only at $12.00 per hour would earnings alone do it. In this case, using the OPM would overstate the anti-poverty effect.

3. The linking was made possible by the data resources and documentation of the IPUMS-CPS at the Minnesota Population Center and the University of Minnesota (Ruggles et al. 2015; Flood et al. 2017).

4. Neumark and Wascher’s work on the EITC is an important exception (2011). Using state-level variations in the EITC and minimum wages, they find that higher minimum wage rates combined with larger EITC supplement rates raise the labor supply of low-skilled mothers and lower the proportion of female-headed households with market earnings below the poverty line.

5. Although we believe the SPM provides a better measurement of disposable income, using the SPM requires either using the general measure published by the U.S. Census Bureau, which is functionally a multiple of the OPM, or making additional assumptions about geographic location, medical out-of-pocket expenses, and work expenses. We believe the former approach adds little and the latter would complicate rather than clarify our analysis.

6. Reports from low-wage working parents subject to the Seattle Minimum Wage show that workers are aware of these effects. Respondents reported worrying about the loss of income supports associated with increased hourly wages (Seattle Minimum Wage Study 2016a).

7. We make these claims based on our experience evaluating the Seattle Minimum Wage Ordinance. Although it may seem counterintuitive that employers would raise wages in advance of a mandate taking effect, we observed this with both survey and administrative data in Seattle (Seattle Minimum Wage Study Team 2016a, 2016b). In interviews, some employers explained they raised wages earlier than they had to because of ongoing practices (for instance, giving raises on employees’ anniversaries) or to stay ahead of market forces (Seattle Minimum Wage Study Team 2017). We believe confusion also plays a role: when asked what minimum wage applied to their firms, 21 percent of small firms responded incorrectly, most commonly over-estimating (Seattle Minimum Wage Study Team 2016a). Close to 10 percent of the surveyed employers thought that they had to pay $15 per hour as of April 1, 2015, even though that amount would not phase in for at least two more years.

8. In comparison, Isabel Sawhill and Quentin Karpilow (2014) assume no disemployment effects in their model of a $10.10 minimum wage and the Congressional Budget Office (2014) estimates a 1.5 percent disemployment effect on a minimum wage increase of 39.3 percent, an effective labor demand elasticity of −0.039.

## APPENDIX

**Table A1.** Earnings Plus Taxes and Select Transfers, One-Adult Households

	One Part-Time Worker				One Full-Time Worker			
Hourly Wage	7.25	10.15	12	15	7.25	10.15	12	15
**No children**								
Annual earnings	7,250	10,150	12,000	15,000	14,500	20,300	24,000	30,000
+ Taxes	7,201	9,736	11,138	13,388	13,005	17,718	20,580	25,221
+ SNAP	9,413	11,528	12,450	14,000	13,705	17,846	20,580	25,221
+ Housing and childcare assistance	18,281	19,520	19,890	20,540	20,401	22,802	24,420	27,261
**One child**								
Annual earnings	7,250	10,150	12,000	15,000	14,500	20,300	24,000	30,000
+ Taxes	9,798	13,747	15,455	18,226	17,764	22,745	25,505	29,756
+ SNAP	13,994	17,579	18,807	21,074	20,744	24,213	26,481	30,136
+ Housing and childcare assistance	28,625	31,346	32,022	32,789	34,520	36,429	36,549	35,752
**Two children**								
Annual earnings	7,250	10,150	12,000	15,000	14,500	20,300	24,000	30,000
+ Taxes	10,233	14,507	17,232	21,225	20,688	25,875	28,495	32,473
+ SNAP	16,269	20,179	22,424	25,637	25,232	29,175	30,843	33,789
+ Housing and childcare assistance	34,607	37,652	39,346	41,658	44,502	46,285	46,849	47,161
**Three children**								
Annual earnings	7,250	10,150	12,000	15,000	14,500	20,300	24,000	30,000
+ Taxes	10,595	15,014	17,832	21,922	21,385	27,167	30,209	34,251
+ SNAP	18,315	22,410	24,748	28,058	27,653	32,199	34,281	36,755
+ Housing and childcare assistance	39,943	43,173	44,960	47,369	51,262	53,648	54,626	55,300

*Source:* Authors’ calculations.

*Note:* 2015 Official Poverty Thresholds, which vary by household size and number of children, are as follows: $12,331 (1 adult), $16,337 (1 adult, 1 child), $19,096 (1 adult, 2 children), $24,120 (1 adult, 3 children), $15,871 (2 adults), $19,078 (2 adults, 1 child), $24,036 (2 adults, 2 children), $28,286 (2 adults, 3 children).

**Table A2.** Earnings Plus Taxes and Select Transfers, Two-Adult Households

	One Full-Time Worker				One Full-Time + One Part-Time Worker			
Hourly Wage	7.25	10.15	12	15	7.25	10.15	12	15
**No children**								
Annual earnings	14,500	20,300	24,000	30,000	21,750	30,450	36,000	45,000
+ Taxes	13,845	18,757	21,834	26,775	19,981	27,146	31,716	38,840
+ SNAP	16,469	19,869	22,190	26,839	20,725	27,210	31,716	38,840
+ Housing and childcare assistance	23,165	24,825	26,030	28,879	25,237	29,118	31,956	38,840
**One child**								
Annual earnings	14,500	20,300	24,000	30,000	21,750	30,450	36,000	45,000
+ Taxes	17,764	23,120	26,495	30,553	24,459	30,852	34,535	40,448
+ SNAP	22,236	26,080	28,495	31,033	27,391	32,156	35,631	40,448
+ Housing and childcare assistance	29,124	31,408	32,719	33,805	37,258	38,420	38,292	40,448
**Two children**								
Annual earnings	14,500	20,300	24,000	30,000	21,750	30,450	36,000	45,000
+ Taxes	20,688	26,319	29,681	33,839	27,658	34,115	37,516	43,033
+ SNAP	26,876	30,991	33,397	35,991	32,306	36,507	39,068	43,729
+ Housing and childcare assistance	33,860	36,235	37,525	38,319	45,880	47,464	47,440	46,436
**Three children**								
Annual earnings	14,500	20,300	24,000	30,000	21,750	30,450	36,000	45,000
+ Taxes	21,385	27,611	31,378	35,656	29,168	35,977	39,618	45,135
+ SNAP	29,193	33,907	36,714	39,428	35,436	39,989	42,190	46,699
+ Housing and childcare assistance	36,321	39,295	40,986	41,900	52,300	54,236	54,782	55,414

*Source:* Authors’ calculations.

*Note:* 2015 Official Poverty Thresholds, which vary by household size and number of children, are as follows: $12,331 (1 adult), $16,337 (1 adult, 1 child), $19,096 (1 adult, 2 children), $24,120 (1 adult, 3 children), $15,871 (2 adults), $19,078 (2 adults, 1 child), $24,036 (2 adults, 2 children), $28,286 (2 adults, 3 children).
