# Supplementary material for: Coupling a Federal Minimum Wage Hike with Public Investments to Make Work Pay and Reduce Poverty
Source: RSF. Author manuscript; Available in PMC 2021 Jul 18. (PMC8286698; doi:10.7758/rsf.2018.4.3.02)
Supplement: Appendix [file NIHMS1588854-supplement-Appendix.pdf]

(ONLINE) Appendix. Assumptions for Simulations in Figure 1, Figure 2, and Appendix Figure 1

Figure 1 and Appendix Figure 1

Means-test benefits in figure 1 and appendix figure 1 were calculated for a one adult, two child household and a two adult, three child household at earning levels ranging from $0 to $60,000 (in $500 increments) using the methods described below.

Value of Childcare Subsidy

The value of the childcare subsidy was calculated by determining the market cost of childcare for each type of household, subtracting any copay the household paid, and adding in the value of the Child and Dependent Care Credit from the household's estimated federal taxes. The market cost of childcare was determined using information collected by Child Care Aware (http://www.usa.childcareaware.org/advocacy-public-policy/resources/reports-and-research/costofcare/). For 2014, Child Care Aware reported average annual childcare costs by state, age of child (i.e., infant, four-year-old and school age), and type of childcare (i.e., childcare center or family childcare). Using information for each state, we calculated a median U.S. annual cost of childcare in a childcare center for each child age group. In order to calculate the total market cost of childcare for all children in each type of family, we made assumptions about the ages of their children and summed the costs for those children. Children living in a one adult, two child household were four years old and school age. Two adult, three child households had one four-year-old and two school-age children.

For families at income levels rendering them eligible for a childcare subsidy, we calculated their copays using the Washington State DSHS Child Subsidy Program copay calculation table. This table lists monthly copays by household size and income level. We multiplied the appropriate monthly copays by twelve to determine families' annual copay.

Child and Dependent Care Credits for 2016 were determined for each income group using the National Bureau of Economic Research's TAXSIM program (http://users.nber.org/~taxsim/taxsim-calc9/index.html). This program calculates federal (and state, if desired) income tax liabilities from survey data, and the output includes values of the Child and Dependent Care Credit and other credits (e.g., Earned Income Tax Credit, Child Tax Credit). For a household consisting of one adult and two children, we set marital status to "head of household." For a household with two adults and three children, we set marital status to "joint." We took exemptions for two (or three) dependents under the age of seventeen. We assumed the household had no income other than earned income. For household consisting of two adults, we split the earned income between them. We reported annual child care expenses for families that were calculated using the methods described above (families paid copays if they were eligible for a childcare subsidy and market value otherwise).

Earned Income Tax Credit (EITC)

EITC values for 2016 were calculated for each income group using the National Bureau of Economic Research's TAXSIM program and the same assumptions used in the calculation of the Child and Dependent Care Credit (see "Value of Childcare Subsidy" section).

Child Tax Credit (CTC)

CTC values for 2016 were calculated for each income group using the National Bureau of Economic Research's TAXSIM program and the same assumptions used in the calculation of the Child and Dependent Care Credit (see "Value of Childcare Subsidy" section). TAXSIM reports the CTC on two lines of output – the first labeled "Child Tax Credit," and the second labeled "Refundable Part." The CTC we report is the sum of these two lines.

Supplemental Nutrition Assistance Program (SNAP)

SNAP benefit calculators are available for some states, but not at the federal level.. We selected calculators from three states: Washington, Oregon and North Dakota. We determined SNAP benefits for a one adult, two child (or three-person) household and for a two adult, three child (or five-person) household in each state, and averaged the benefit level across the three. We assumed the households had only earned income from jobs (no

TANF, SSI, self-employment, etc.). For expenses, we included rent, child (or dependent) care, and utilities. Across the three calculators, rent was set at 30 percent of earned income. Child care expenses were set at the copay shown in the Washington State DSHS Child Subsidy Program copay calculation table. We assumed families ineligible for a childcare subsidy paid full market value for childcare, which was calculated as described above. Each SNAP benefits calculator collected information about utilities in different ways. For Washington, we assumed the household paid “heating or cooling,” “electric not used for heating,” and “telephone.” For Oregon, we assumed the household paid for “heat separate from rent.” For North Dakota, we assumed the household paid $191.00 in utilities, which was selected from a drop down menu with five utility standard choices. These three calculators can be found at the following web addresses:

Washington: http://foodhelp.wa.gov/bf_benefit_estimator.htm

Oregon: https://aix-xweb1p.state.or.us/caf_xweb/SNAP_Estimate/actCalcEstimate.cfm

North Dakota: https://www.nd.gov/dhs/snap/simplecalculator.aspx

These calculators provide a monthly SNAP benefit. To determine the annual benefit, we multiple each wage group’s monthly benefit by twelve.

Value of Housing Subsidy

To determine the value of a household’s housing subsidy, we used the Income, Rent & Utility Calculation Worksheet from the Indiana Housing & Community Development Authority (see: http://www.in.gov/ihcda/files/Copy_of_Income__Rent_Calculation_Form(1).xls). This worksheet is based on federal HUD rules. We assumed the household had only earned income, and child care expenses were calculated using the Washington State DSHS Child Subsidy Program copay calculation table or, for household ineligible for childcare subsidies, the authors calculations of U.S. median market cost of childcare using 2014 data from Child Care Aware (see “Value of Childcare Subsidy” section for details on the calculation of this value). For all wage groups, monthly rent was set to $920, the median gross rent for all U.S. households, and we assumed utilities were included with rent. We used Social Explorer (https://www.socialexplorer.com/) and data

from the 2014 American Community Survey 5-year estimates to determine the U.S. median gross rent (see also: https://www.census.gov/content/dam/Census/newsroom/releases/2015/gross_rent.pdf). We then multiplied the monthly value of the housing subsidy by twelve to determine the annual value.

Figure 2 and Appendix Table 2

The income and marginal tax rate values in figure 2 and appendix table 1 were calculated as follows.

Earnings

For part-time workers living in one adult households, we calculated annual earnings by multiplying the designated hourly wage (e.g., $7.25, $10.15) by 1,000 hours. For one adult and two households comprised of one full-time worker, we multiplied the hourly wage by 2,000 hours. For two adult household comprised of one full-time worker and one part-time worker, we multiplied the hourly wage by 3,000 hours.

Net Federal Taxes

As with the tax credit calculation described above, we used the TAXSIM program to calculate net 2016 federal taxes for 2016 for each wage group. For household consisting of one adult and one, two or three children, we set marital status to "head of household" and took exemptions for the corresponding number of dependents under the age of seventeen. We assumed the household had no income other than earned income. We reported subsidized childcare expenses for families calculated based on Washington State rules, as described above, in the event that they qualified for the federal Child and Dependent Care Credit. For one adult and two adult households with no children and for two adult households with one worker, we assumed no childcare costs.

SNAP, Housing, and Child Care Assistance

With a few exceptions, the food, housing, and childcare assistance calculations in figure 2 and appendix table 1 followed the format used for figure 1 and appendix figure 1. We used the same assumptions and three-state averaging procedure to calculate SNAP benefits. Similarly, the figure 2 and appendix table 2 housing subsidy values were calculated as described above, applying the Washington State childcare subsidies for households that contained children and only working adults (i.e. no childcare costs for childless and two-adult, one-worker

households). Finally, we calculated the value of the childcare voucher by determining the market cost of childcare and subtracting the family's copay using the above assumptions about market cost and the Washington State subsidy. However, for the purpose of figure 2 and appendix table 2, the Child and Dependent Care Credit was included under net federal taxes rather than as part of the childcare subsidy.

Appendix Table 3. Effective Marginal Tax Rates based on Table 3 Simulations, One-Adult Households

	Estimated Marginal Tax Rates (in percent) for Wage Increases of:		
	$7.25 → $10.15	$10.15 → $12.00	$12.00 → $15.00
0 Children			
Part-time hours			
Net federal taxes + SNAP	27.1	50.1	48.4
...plus housing and child care	57.3	80	78.4
Full-time hours			
Net federal taxes + SNAP	28.6	26.1	22.7
...plus housing and child care	58.6	56.3	52.7
1 Child			
Part-time hours	-23.6	33.6	24.5
Net federal taxes + SNAP	6.2	63.4	74.5
...plus housing and child care			
Full-time hours			
Net federal taxes + SNAP	40.2	38.7	39.1
...plus housing and child care	67.1	96.8	113.3
2 Children			
Part-time hours			
Net federal taxes + SNAP	-34.8	-21.4	-7.1
...plus housing and child care	-5	8.5	22.9
Full-time hours			
Net federal taxes + SNAP	32	54.9	50.9
...plus housing and child care	69.3	84.8	94.8
3 Children			
Part-time hours			
Net federal taxes + SNAP	-41.2	-26.4	-10.3
...plus housing and child care	-11.4	3.4	19.7
Full-time hours			
Net federal taxes + SNAP	21.6	43.7	58.8
...plus housing and child care	58.9	73.5	88.8

Sources: Authors' calculations.

Appendix Table 4. Effective Marginal Tax Rates based on Table 3 Simulations, Two-Adult Households

	Estimated Marginal Tax Rates (in percent) for Wage Increases of:		
	$7.25 → $10.15	$10.15 → $12.00	$12.00 → $15.00
0 Children			
1 Full-time Worker			
Net federal taxes + SNAP	41.4	37.3	22.5
...plus housing and child care	71.4	67.4	52.5
1 Full-time + 1 Part-time Worker			
Net federal taxes + SNAP	25.5	18.8	20.9
...plus housing and child care	55.4	48.9	23.5
1 Child			
1 Full-time Worker			
Net federal taxes + SNAP	33.7	34.7	57.7
...plus housing and child care	60.6	64.6	81.9
1 Full-time + 1 Part-time Worker			
Net federal taxes + SNAP	45.2	37.4	46.5
...plus housing and child care	86.7	102.3	76.1
2 Children			
1 Full-time Worker			
Net federal taxes + SNAP	29	35	56.8
...plus housing and child care	59	65.1	86.8
1 Full-time + 1 Part-time Worker			
Net federal taxes + SNAP	51.7	53.8	48.2
...plus housing and child care	81.8	100.4	111.2
3 Children			
1 Full-time Worker			
Net federal taxes + SNAP	18.7	24.1	54.8
...plus housing and child care	48.7	54.3	84.8
1 Full-time + 1 Part-time Worker			
Net federal taxes + SNAP	47.7	60.3	49.9
...plus housing and child care	77.7	90.2	93

Sources: Authors' calculations.
